# Syphilis/HIV Early Rapid Response (SHIVER): Community-Driven Point-of-Care Testing (POCT) Across Diverse Sites in Saskatchewan, Canada

**DOI:** 10.3390/diagnostics16213445

**Published:** 2026-10-22

**Authors:** Cara Spence, Visna Rampersad, Nicole Williamson, Srinivasa Tejaswini Banna, Noreen Reed, Lindsay Iron, Jessica Minon, Beverly Wudel, Claire Allen, Kristin McBain, Rick Galli, Sean B. Rourke, Stuart Skinner

**Affiliations:** 1 Department of Medicine, University of Saskatchewan, Saskatoon, SK S7N 0X8, Canada; srinivasa.banna@usask.ca (S.T.B.); noreenreed@acn104.ca (N.R.); beverly.wudel@gmail.com (B.W.); stuart.skinner@usask.ca (S.S.); 2 Indigenous Services Canada–Saskatchewan Region, Regina, SK S4P 2B6, Canada; 3 Wellness Wheel Medical Clinic, Regina, SK S4T 0L6, Canada; nikki.williamson@wellnesswheelclinic.ca (N.W.); gza479@mail.usask.ca (C.A.); 4 Meadow Lake Tribal Council, Meadow Lake, SK S9X 1T8, Canada; lindsay.iron@mltc.net; 5 Saskatchewan Health Authority, 1440 14th Avenue, Regina, SK S4P 0W5, Canada; jessica.minion@saskhealthauthority.ca; 6 MAP Centre for Urban Health Solutions, St. Michael’s Hospital (Unity Health Toronto), Toronto, ON M5B 1W8, Canada; kristin.maclennan@unityhealth.to (K.M.); rgalli@summitdx.net (R.G.); sean.rourke@utoronto.ca (S.B.R.); 7 Department of Psychiatry, University of Toronto, 250 College Street, Toronto, ON M5T 1R8, Canada

**Keywords:** health equity, point-of-care testing (POCT), syphilis, HIV, communityoutreach, access to care, model of care

## Abstract

**Background:** Saskatchewan faces a growing public health challenge, with outbreaks of infectious syphilis and a continued increase in new HIV diagnoses. Rural, remote, and underserved populations experience disproportionate burdens due to barriers to timely testing, diagnosis, treatment, and linkage to care. The Syphilis/HIV Early Rapid Response (SHIVER) project implemented an innovative point-of-care testing (POCT) and treatment model to improve access to rapid diagnosis, immediate treatment, and care for individuals in inner-city, rural, remote, and underserved communities across Saskatchewan, Canada. **Methods:** Individuals aged ≥16 years were offered POCT for syphilis and/or HIV based on non-random criteria. Standard serology was collected according to provincial guidelines. Participants with a positive syphilis POCT result were offered immediate treatment, when appropriate, and follow-up testing. Those with a positive HIV POCT received counselling, confirmatory testing, and expedited linkage to care. **Results:** Between February 2023 and November 2024, 1797 participants were enrolled. Syphilis POCT was completed by 1781 participants, including 319 (17.9%) first-time testers. HIV POCT was completed by 1387 participants, including 141 (10.2%) first-time testers. The program achieved a diagnostic yield of 16.45% for syphilis and 3.39% for HIV. Integrating dual POCT in a single encounter increased testing opportunity, improved case detection, and minimized procedural burden for participants. **Conclusions:** This flexible POCT model successfully expanded access to testing, immediate treatment, and timely linkage to care for populations at the highest risk of syphilis and HIV. Its emphasis on community partnerships and culturally safe service delivery supports a scalable model that can improve access to infectious disease care in underserved communities. Trial Registration: ClinicalTrials.gov (NCT05863117) Date of Registration: 19 April 2023.

## 1. Introduction

Syphilis and HIV (Human Immunodeficiency Virus) are both significant sexually transmitted infections (STIs) that, left untreated, can lead to serious health consequences, including transmission in utero during pregnancy and even death [1,2]. Although caused by different pathogens, both syphilis and HIV are primarily transmitted through sexual contact and share common risk factors [3]. Importantly, both infections can be effectively prevented and treated [4,5], and early diagnosis enables timely medical intervention and improved health outcomes [6]. Routine screening is therefore critical for early detection, controlling transmission, and ensuring prompt treatment [7].

Saskatchewan currently faces a critical public health challenge, with the worst outbreaks of infectious syphilis in Canada since 1948 and a substantial increase in new HIV diagnoses [8]. The province spans approximately 577,060 km^2^ and has a relatively low population density, with around 1.2 million residents or 2 per m^2^. Nearly 45% of the population resides in the two main urban centers, Saskatoon and Regina, while 37% live in rural and remote areas, and the remaining 18% are spread across smaller communities [9]. Indigenous communities are predominantly in rural and remote northern regions, though there is also a significant Indigenous presence in urban areas [9].

Indigenous peoples in Canada, including First Nations, Métis, and Inuit, comprise approximately 16% of Saskatchewan’s population, compared to just 5% nationally [9]. The province is party to intergovernmental agreements and is subject to treaties and other agreements with the federal government intended to support equitable resource sharing and access to healthcare and education. Despite these agreements, significant social and health disparities persist in Indigenous communities [10,11,12]. Healthcare systems often struggle to effectively partner with Indigenous communities and organizations to deliver care where it is needed most. This challenge is particularly acute in many rural and remote communities, which face limited infrastructure and resources. These barriers cause delays or lack of access to essential services, including emergency, primary, and specialist care, worsening chronic conditions and health inequities. Overall, the provincial healthcare system continues to face significant challenges in providing equitable care and accessible services to Indigenous communities and rural and remote populations and generally lacks culturally responsive care that respects Indigenous cultural values and healing practices. In response, such care is more commonly delivered through community-based and Indigenous-led organizations.

In 2023, the national rate of infectious syphilis was 30.5 per 100,000 population. However, Saskatchewan experienced a dramatically higher rate of 159.3 per 100,000, the highest among all provinces and territories [13]. Even more concerning is the 1213% increase in infectious syphilis diagnoses in Saskatchewan since 2018, signaling a rapidly worsening epidemic [13]. In comparison, the national rate of new HIV diagnoses in 2023 was 6.1 per 100,000 population, while Saskatchewan reported a significantly elevated rate of 19.4 per 100,000, more than three times the national average and the highest in the country [14]. These alarming trends highlight the urgent need for more accessible and responsive healthcare solutions in underserved areas. One promising approach explored in this study is point-of-care testing (POCT), which enables rapid, on-site testing and treatment in community-based and non-traditional medical settings, helping to deliver care where it is most needed.

POCT is widely available for diagnostic and treatment purposes in many countries. In Canada, a scoping review found that between 81% and 97% of Canadians prefer POCT over traditional laboratory testing, with 96% to 100% reporting high satisfaction with the process [15]. Studies in British Columbia and Ontario have demonstrated POCT’s effectiveness in reaching undiagnosed individuals, particularly for HIV [16,17]. The multiplex Point-of-care Tests for Syphilis and HIV (PoSH) Study, launched in August 2020, evaluated two POCT devices among over 1500 participants across clinical and community settings in Edmonton and Northern Alberta. These included community clinics, emergency departments, urgent care centers, correctional facilities, and a First Nations community. The study demonstrated the clinical utility of POCT in identifying previously undiagnosed individuals living with syphilis and/or HIV, while also facilitating rapid linkage to treatment and care, with tests providing results in under five minutes using a fingerstick blood specimen [18]. The study found that both devices were over 98% accurate in identifying active syphilis and 100% accurate in detecting HIV infection, leading to the approval of the tests for clinical use in Canada.

With syphilis and HIV cases rapidly increasing in Saskatchewan, POCT offers an important opportunity to provide immediate, rapid access to testing and care in rural and remote communities, as well as non-traditional settings to provide access to those most in need. Rooted in partnerships, flexibility, and responsiveness, the Syphilis/HIV Early Rapid Response (SHIVER) innovative care model was designed to bring POCT to community settings with the aim of bridging healthcare gaps, promoting early diagnosis, and reducing the burden of infection in underserved and marginalized populations by delivering testing and treatment in trusted, accessible environments. The findings from this approach could play a pivotal role in shaping future healthcare strategies, especially in rural, remote, and Indigenous communities where timely access to care is critical.

## 2. Materials and Methods

### 2.1. Study Aim

Using the RE-AIM Implementation Science Framework [19,20], this study was designed to be mobile and adaptable, delivering syphilis and HIV POCT across diverse communities through trusted community partnerships and spaces. The approach was deeply community-led, with nursing teams often directing service delivery by determining locations and procedures, while the study team provided support and facilitation. The collaborative model included immediate access to an infectious disease physician for consultation, which supported nursing teams in delivering confident, high-quality care. Testing and treatment were provided in non-traditional care settings, such as cargo vans parked outside local stores and at powwows, community and healing events, demonstrating how the program met individuals in the everyday spaces they attend. Participants were offered POCT for syphilis, HIV, or both, with opportunities for immediate treatment and linkage or re-linkage to care, accompanied by culturally relevant educational materials. Through this responsive, community-enabled model, the study aimed to enhance access to care and reduce health disparities. 

### 2.2. Study Design

This study was a prospective, open-label, single group assignment, intervention model evaluating decentralized point-of-care testing (POCT) for HIV and syphilis across real-world community outreach and clinical settings in Saskatchewan. Due to the nature of open-label implementation design and the real-world operational model of immediate point-of-care testing, no blinding or comparative allocation was utilized. Participants, field personnel administering the rapid tests, and individuals recording outcome data were fully aware of the study condition and immediate diagnostic test results. A key aspect of the study design was the development of a community-level strategy, created collaboratively with Indigenous healthcare providers and community leaders to ensure cultural relevance and community appropriateness. A diverse team of health professionals, including outreach workers and peer supports, provided a comprehensive approach to care and support for all participants. Before enrollment, participants provided written or verbal consent. Enrollment took place between February 2023 and November 2024, during which participants received a $20 honorarium in recognition of reciprocity for their contribution. The demographic and geographic profiles of participants were examined, with analyses stratified by age, location and ethnicity, and by syphilis and HIV POCT. The study received research ethics and operational approval from the Saskatchewan Health Authority. The unit of analysis was the individual participant. All operational outcomes such as test selection preferences, and diagnostic yields were measured and reported at the individual level using descriptive statistics (counts and percentages).

### 2.3. Setting

The study was conducted across a diverse range of sites throughout Saskatchewan, encompassing urban, rural, and remote locations. Urban sites in Regina and Saskatoon included key community organizations and healthcare facilities, including the All Nations Hope Network, AIDS Programs South Saskatchewan, the John Howard Society of Saskatchewan, the Labor and Birth Unit at Regina General Hospital, Queen City Wellness Pharmacy, Street Culture Youth Shelter, Street Worker’s Advocacy Project, Wellness Wheel Medical Clinic, Saskatoon Westside Community Clinic, and a correctional facility. Rural and remote sites covered several towns, including Kamsack, Moose Jaw, and Yorkton. With the invitation and agreement of community leadership, the study was also extended to First Nation communities across the province (see Supplementary Figure S1).

### 2.4. Study Participants

Eligibility for the study was open to individuals aged 16 and older who could provide informed consent and were willing to undergo syphilis and/or HIV screening. People with a history of syphilis and those living with HIV were also eligible to participate. Participants were offered POCT for syphilis and/or HIV, alongside serological testing, to gain a comprehensive understanding of their health status. Those with a positive POCT result for syphilis received immediate treatment as appropriate, with follow-up testing arranged. For participants with a positive HIV POCT result, counselling and confirmatory testing were provided promptly, followed by rapid linkage to care to ensure timely support and management. No participants were lost to follow-up and treatment was completed, as appropriate. All eligible individuals who expressed interest in participating and provided informed consent were prospectively enrolled at each study site. This strategy minimized potential selection bias and avoided selective sampling. No systematic random sampling was used.

### 2.5. Test Methods

The first, the Reveal^®^ Rapid Syphilis Test (MedMira Inc., Halifax, NS, Canada), targeted syphilis exclusively, producing results in three minutes by detecting syphilis antibodies, suited for participants interested only in syphilis screening. Second, the INSTI Multiplex^®^ HIV & Syphilis Test (bioLytical Laboratories Inc., Richmond, BC, Canada) simultaneously screened for HIV and syphilis antibodies, delivering results for both infections in under two minutes—convenient for dual testing in a single visit. Third, the INSTI^®^ HIV-1/HIV-2 Antibody Test (bioLytical Laboratories Inc., Richmond, BC, Canada) provided rapid HIV screening results in one minute by detecting HIV antibodies, ideal for those seeking HIV testing alone.

### 2.6. Data Collection

All POCT visits were recorded in REDCap™, a secure web-based data collection platform, as a standardized data collection tool across study sites to ensure consistent and accurate tracking of participant information. Participants were asked detailed questions about their testing history, including whether they had previously been tested for syphilis or HIV, the timing of their last test, known results, and any past treatment received. Following testing, participants were also asked if they would undergo POCT again. Key sociodemographic information, including age, sex, and ethnicity, was also collected to provide a clearer understanding of the participant population.

### 2.7. Data Analysis

Descriptive statistics including counts and percentages were calculated to summarize participant demographic characteristics by various subgroups, POCT option selection (dual HIV/syphilis vs. single pathogen testing) and diagnostic yields. In alignment with implementation-focused design, no comparative inferential statistical testing was performed. No formal hypothesis testing or post-hoc statistical adjustments were applied across subgroups. Data management and descriptive statistics were performed through pivot tables using Microsoft Excel.

## 3. Results

### 3.1. Participant Demographics

The overall analysis included 1797 participants, of whom 1781 completed syphilis POCT and 1387 underwent HIV POCT (Table 1). Females comprised approximately half of the sample, representing 50.20% in the syphilis analysis (n = 894), 49.75% in the HIV analysis (n = 690), and 50.19% overall (n = 902). Males accounted for 49.58% in the syphilis group (n = 883), 50.04% in the HIV group (n = 694), and 49.58% overall (n = 891). A very small proportion identified as others, comprising 0.22% across all analyses.

Most participants were between 19 and 39 years old, making up 50.98% in the syphilis group (n = 908), 52.63% in the HIV group (n = 730), and 50.86% overall (n = 914). Those aged 40 to 59 years represented roughly 36% across all groups, while 16- to 18-year-olds accounted for 4.32% in the syphilis group (n = 77) and 2.81% in the HIV group (n = 39). Participants aged 60 and over ranged from 8.37% in the syphilis group (n = 149) to 9.16% in the HIV group (n = 127). Regarding ethnicity, the majority identified as Indigenous, comprising 83.94% in the syphilis analysis (n = 1495), 83.35% in the HIV analysis (n = 1156), and 83.86% overall (n = 1507). White participants accounted for approximately 12.69% to 13.63%, while those identifying other ethnicities made up about 2.45%. Data were missing for less than 1% of participants.

In partnership with First Nation communities, 36.83% of participants were tested within their communities (n = 656), 27.79% at community-based organizations (n = 495), and 23.47% at pharmacy sites (n = 418). Smaller proportions were tested at healthcare clinics or facilities (5.39%, n = 96), rural towns (5.50%, n = 98), and correctional facilities (1.01%, n = 18). Overall, 53.37% (n = 959) of participants were from urban areas, while 46.63% (n = 838) resided in rural and remote locations. The vast majority of participants (97.05%, n = 1744) indicated they would be willing to undergo POCT again in the future. No adverse events were reported.

### 3.2. POCT Outcomes

Among the 1781 participants included in the syphilis analysis (Table 2), self-reported syphilis status showed that 15.61% were positive, 75.97% negative, and 8.42% unknown. This distribution was similar across geographic areas, with 15.61% positivity among urban participants and 15.33% among those from rural and remote locations. Most participants (73.55%) reported having previously been tested for syphilis, with comparable testing rates in urban (75.05%) and rural/remote (71.86%) populations. Approximately 17.91% had never been tested, while 8.53% were unsure of their testing history. Regarding treatment history, 10.61% reported having been previously treated for syphilis, with a slightly higher proportion in rural/remote participants (12.57%) compared to urban (8.88%). The vast majority (89.11%) reported never having been treated. POCT results indicated that 16.45% of participants tested reactive overall, with a higher proportion in urban areas (19.43%) compared to rural/remote areas (12.56%). Notably, of all the participants with reactive syphilis POCT results (n = 293), n = 137 were previously undiagnosed, while n = 156 had previously self-reported a previous syphilis diagnosis. Non-reactive results accounted for 82.82% overall, while invalid results were rare at 0.73%. Syphilis treatment was provided during the visit to 6.12% of participants, more commonly in urban sites (7.40%) than in rural/remote locations (4.67%).

Among the 1387 participants included in the HIV analysis (Table 3), 1.87% self-reported as HIV-positive, with a higher proportion in urban areas (2.65%) compared to rural and remote regions (0.82%). Most participants reported being HIV-negative (92.36% overall), with similarly high rates in urban (93.82%) and rural/remote (90.49%) areas. A larger share of rural and remote participants (8.69%) reported unknown HIV status compared to urban participants (3.47%).

Regarding HIV testing history, 84.93% of participants had previously tested, with higher rates in urban areas (89.64%) than in rural and remote areas (78.20%). Conversely, a greater proportion of rural and remote participants (13.77%) had never been tested. Among those with reported HIV POCT test results, the majority of tests were non-reactive (95.53%), and invalid results were infrequent, occurring in 1.08% of the tests overall.

Of the total participants who consented to testing (N = 1797), 75.68% (n = 1360) selected the multiplex INSTI HIV/Syphilis dual kit, while 22.82% (n = 410) opted for syphilis-only testing by MedMira. The remaining participants chose single-disease screening via the INSTI HIV-only test (0.89%, n = 16) or a sequential testing of HIV then syphilis (0.61%, n = 11). This distribution of the POCT test chosen indicated a participant preference for integrated diagnostic services (dual testing). Approximately 75% of the cohort selected a multiplex platform to maximize diagnostic insights within a single clinical encounter, while minimizing procedural discomfort (Table 4).

### 3.3. Diagnostic Yield Among All Tested

Diagnostic yield is defined as the proportion of valid point-of-care tests that yield a conclusive reactive result in the total screening cohort [21]. Unlike true diagnostic accuracy, which evaluates a tool’s definitive performance parameters against a clinical gold standard [22], diagnostic yield serves as a practical implementation metric that quantifies real-world case-finding efficiency in community-based outreach settings [21].

### 3.4. Syphilis Diagnostic Yield

Out of 1781 administered syphilis point-of-care tests, n = 13 tests were invalid. Among the remaining valid screening encounters (n = 1768), the program achieved an overall syphilis diagnostic yield of 16.45% (n = 293 reactive cases). When analyzed by gender, the diagnostic yield was higher among female participants at 18.12% (n = 162 out of 894) compared to 14.84% (n = 131 out of 883) among male participants (Table 5).

### 3.5. HIV Diagnostic Yield

Of 1387 administered HIV point-of-care tests, n = 15 were invalid. Among the remaining valid screening encounters (n = 1372), the program achieved an overall HIV diagnostic yield of 3.39% (n = 47 reactive cases). By sex, the diagnostic yield was slightly higher among female participants at 3.62% (n = 25 of 690) than among male participants at 3.17% (n = 22 of 694) (Table 6).

## 4. Discussion

### 4.1. Participants

This study provided a comprehensive demographic profile of nearly 1800 participants who underwent POCT for syphilis and HIV across diverse community and geographical settings. The roughly equal representation of males and females, along with a small proportion identifying as other, demonstrated an inclusive sampling approach that enhanced the generalizability of the findings across sexes. The age distribution was notably concentrated among individuals aged 19 to 39, aligning with epidemiological evidence indicating higher STI prevalence among younger adults [23,24], emphasizing the urgent need to target preventive and diagnostic efforts toward this demographic. Additionally, high rates among participants aged 40 to 59 highlighted that STI risk and screening remained relevant beyond younger populations, reinforcing the importance of including a broad age range in public health strategies.

In terms of ethnicity, the study had strong representation of Indigenous participants, who comprised approximately 84% of the sample. This underscored the impact of meaningful engagement and leadership from Indigenous communities in healthcare delivery in engaging the community. The Canadian Institutes for Health Research, in partnership with the Public Health Agency of Canada and Indigenous Services Canada, has identified Indigenous-led and community-partnered delivery as key to successful engagement, where building trust within communities and providing services in trusted, community-owned spaces meaningfully improves testing uptake, recruitment, and retention among Indigenous populations [25]. Recognizing the disproportionate burden these infections have historically placed on Indigenous populations [26], the study design and targeted testing efforts reflected a commitment to culturally relevant, community-based approaches that prioritized Indigenous knowledge, resilience, and self-determination. In terms of intervention characteristics and scalability, the study used ready-to-use rapid test kits that are already widely available. Because the testing process is simple and provides results in just a few minutes, this approach can easily be used in other real-world outreach programs where getting fast results is vital to keeping people engaged. Results from this interventional study also informed the development of a multi-year action plan strategy to address sexually transmitted and blood-borne infections (STBBIs) by the Saskatchewan Ministry of Health [27].

Geographically, the study involved urban and rural participants, with a significant proportion (46.63%) tested in rural and remote regions. This distribution underscored that the approach utilized in the study in providing accessible POCT in areas with limited healthcare resources and of delivering care within familiar, community-based settings is effective in all areas. This finding aligns with Alberta’s rapid POCT program, which was explicitly extended beyond metropolitan zones into rural, remote communities as part of its scale-up strategy, reflecting a recognition that syphilis cases were increasingly occurring in areas where access to services is limited and may result in delays in treatment [28]. Similarly, Ontario’s Syphilis Point of Care Rapid Testing and Immediate Treatment Evaluation (SPRITE) study built the implementation model around non-urban, rural, and remote public health units specifically because of the paucity of evidence regarding the effectiveness of POC tests in non-urban, rural, and remote settings [29]. Partnerships in non-traditional and community-based settings proved essential in delivering care, ensuring equitable healthcare access, and addressing health disparities [25]. The high levels of willingness to be tested, specifically 75% of participants comfortable with receiving both an HIV and syphilis dual test, demonstrate that the context of the care model and the cultural responsiveness of the healthcare team administering the test are key factors in POCT uptake.

### 4.2. POCT Analysis

The analysis of syphilis testing among 1781 participants revealed a substantial burden of infection and high engagement with testing across both urban and rural/remote populations. Self-reported syphilis positivity status was relatively high at 15.6%, with similar rates between urban (15.61%) and rural/remote (15.33%) participants, indicating that syphilis affects diverse geographic areas without significant disparity in self-awareness of infection status. Reactive POCT results were slightly more common in urban areas (19.13%) than in rural/remote regions (13.41%), which may reflect improved detection capabilities, higher risk, and greater access to testing in urban settings. Non-reactive results accounted for the majority (82.8%), while invalid results were rare. These elevated positivity rates underscore the urgent need for sustained public health efforts focused on prevention, routine screening, and timely treatment to reduce transmission and improve health outcomes across all regions.

Testing history for syphilis further highlighted geographic differences: most participants (73.5%) had self-reported being previously tested, with nearly equal rates in urban (75.05%) and rural/remote (71.86%) areas. However, approximately 18% had never been tested, and 8.5% were unsure of their testing history, revealing gaps in testing coverage that may contribute to undiagnosed infections and ongoing transmission. A systematic review conducted by Laprise and Bolster-Foucault (2021) found that barriers included difficulties accessing testing services, fear and stigma surrounding HIV, low risk perception, insufficient patient confidentiality and lack of resources for testing over the 2009–2019 period [30]. Therefore, this should not be interpreted as simple disengagement or lack of concern about sexual health, but as a marker of structural and psychosocial barriers that are prevalent.

Similarly, a recent commentary published by PHAC on HIV self-testing found that individuals reporting never having been tested for HIV tended to be younger and were more likely to belong to racialized minority groups, characteristics that overlap substantially with those facing the greatest barriers to primary care access in Canada [31]. The HIV analysis of 1387 participants revealed important patterns in self-reported status, testing history, and POCT outcomes, with notable differences between urban and rural/remote populations. The overall self-reported HIV positivity rate was low at 1.87% but was significantly higher in urban areas (2.70%) compared to rural and remote regions (0.82%). This urban predominance could reflect a combination of higher transmission rates, greater awareness, or better access to diagnostic services in cities. However, cities are not uniformly high-access environments; they contain both well-resourced populations and also a concentrated, often invisible, group of urban-marginalized individuals (i.e., people experiencing homelessness, unstable housing, or lacking provincial health coverage) for whom conventional urban healthcare infrastructure remains functionally inaccessible [32]. Most participants reported being HIV-negative (over 90% in both urban and rural/remote areas), indicating a generally low self-reported HIV burden. However, the substantially higher proportion of participants with unknown HIV status in rural and remote regions (8.69%) compared to urban areas (3.47%) highlights critical gaps in knowledge and potential access to testing services within these underserved communities were also emphasized, as in the SPRITE study [29]. The incidence of invalid tests was low (1.08%), supporting the consistent performance of the rapid testing method used across settings. 

HIV testing history echoed disparities. While a large majority (84.93%) had previously been tested for HIV, urban participants reported substantially higher prior testing rates (nearly 90%) than those from rural and remote areas (78.20%). Conversely, a higher proportion of rural and remote participants had never been tested (13.77%) than urban residents (10.17%), suggesting persistent barriers to regular testing in less accessible regions. These findings reveal notable geographic disparities in awareness, testing history, and prevalence: urban areas show higher diagnosis and seropositivity rates, while rural and remote regions experience greater unknown status and lower testing uptake. Additionally, diagnostic yield is an important metric for population coverage. By analyzing overall syphilis and HIV diagnostic yields (16.45% and 3.39%, respectively) and laboratory metrics, this study directly accounted for real-world implementation factors, including specimen availability, turnaround time, test robustness, and user-friendliness. These factors collectively contribute to the generation of effective, timely results in a real-world clinical setting but are not usually covered by diagnostic accuracy studies [21].

## 5. Conclusions

By leveraging trusted local spaces, the model established a reproducible, scalable model to reach underserved populations across diverse urban and rural settings and Indigenous communities, addressing not only these STIs but also broader public health challenges in other communities. This responsive and rapid POCT initiative highlights the critical role of community-driven partnerships in reducing health inequalities and promoting well-being across regions and communities. Closing gaps in care requires improving access to testing, treatment, and education in rural and remote areas to ensure early diagnosis and timely linkage to care, ultimately lowering transmission risks and mitigating health disparities.

## Figures and Tables

**Table 1:** Demographic profile of participants.

	**Overall Analysis** **(n = 1797)**		**Syphilis Analysis** **(n = 1781)**		**HIV Analysis** **(n = 1387)**	
	**Count (n)**	**Proportion (%)**	**Count (n)**	**Proportion (%)**	**Count (n)**	**Proportion (%)**
Sex						
Female	902	50.19%	894	50.20%	690	49.75%
Male	891	49.58%	883	49.58%	694	50.04%
Other	4	0.22%	4	0.22%	3	0.22%
Age (years)						
16 to 18	77	4.28%	77	4.32%	39	2.81%
19 to 39	914	50.86%	908	50.98%	730	52.63%
40 to 59	654	36.39%	647	36.33%	491	35.40%
60+	152	8.46%	149	8.37%	127	9.16%
Ethnicity						
Indigenous	1507	83.86%	1495	83.94%	1156	83.35%
White	230	12.80%	226	12.69%	189	13.63%
Other	44	2.45%	44	2.47%	27	1.95%
Unknown	16	0.89%	16	0.90%	15	1.08%
Study Geography						
Urban	959	53.37%	946	53.12%	777	56.02%
Rural & Remote	838	46.63%	835	46.88%	610	43.98%
Study Sites						
Healthcare Clinics/Facilities	96	5.34%	96	5.39%	56	4.04%
Community-Based Organizations	497	27.66%	495	27.79%	416	29.99%
Rural Towns	98	5.45%	98	5.50%	88	6.34%
First Nation Communities	659	36.67%	656	36.83%	451	32.52%
Pharmacy	429	23.87%	418	23.47%	358	25.81%
Correctional Facility	18	1.00%	18	1.01%	18	1.30%

**Table 2:** Syphilis trends among participants by study geography.

	Overall (n = 1781)		Urban (n = 945)		Rural & Remote (n = 655)	
	Count (n)	Proportion (%)	Count (n)	Proportion (%)	Count (n)	Proportion (%)
Self-Reported Status						
Syphilis Positive	278	15.61%	150	15.61%	128	15.33%
Syphilis Negative	1353	75.97%	731	77.27%	622	74.49%
Unknown Status	150	8.42%	65	8.42%	85	10.18%
Self-Reported Syphilis Testing History						
Previously Tested	1310	73.55%	710	75.05%	600	71.86%
Never Tested	319	17.91%	166	17.55%	153	18.32%
Unknown	152	8.53%	70	7.40%	82	9.82%
Self-Reported Syphilis Treatment History						
Previously Treated	189	10.61%	84	8.88%	105	12.57%
Never Treated	1587	89.11%	862	91.12%	725	86.83%
Unknown	5	0.28%	0	0%	5	0.6%
Syphilis POCT Results						
Reactive	293	16.45%	181	19.13%	112	13.41%
Non-reactive	1475	82.82%	760	80.34%	715	85.63%
Invalid	13	0.73%	5	0.53	8	0.96%
Syphilis Treatment Provided During Visit						
Yes	109	6.12%	70	7.40%	39	4.67%
No	1672	93.88%	876	92.60%	796	95.33%

**Table 3:** HIV trends among participants by study geography.

	Overall (n = 1387)		Urban (n = 777)		Rural & Remote (n = 610)	
	Count (n)	Proportion (%)	Count (n)	Proportion (%)	Count (n)	Proportion (%)
Self-Reported Status						
HIV-Positive	26	1.87%	21	2.70%	5	0.82%
HIV-Negative	1281	92.36%	729	93.82%	552	90.49%
Unknown Status	80	5.77%	27	3.47%	53	8.69%
Self-Reported HIV Testing History						
Previously Tested	1178	84.93%	701	84.93%	477	78.20%
Never Tested	141	10.17%	57	10.17%	84	13.77%
Unknown	68	4.90%	19	4.90%	49	8.03%
HIV POCT Results						
Reactive	47	3.39%	39	5.02%	8	1.31%
Non-reactive	1325	95.53%	734	94.47%	591	96.89%
Invalid	15	1.08%	4	0.51%	11	1.80%

**Table 4:** POCT test selection among participants.

Test Type Selected	Percentage of Total Program Uptake	Count of Total Program Uptake (N = 1797)
INSTI HIV/Syphilis Dual	75.68%	1360
MedMira Syphilis Only	22.82%	410
HIV then syphilis	0.61%	11
INSTI HIV Only	0.89%	16

**Table 5:** Syphilis test results and diagnostic yield among participants, overall and by sex.

Sex	Test Result (n%)				Diagnostic Yield % (95% CI)
	Reactive	Non-Reactive	Invalid	Total	
Female	162	728	4	894	18.12 (15.7–20.7)
Male	131	743	9	883	14.84 (12.6–17.3)
* Other		4		4	-
Total	293	1475	13	1781	16.45 (14.8–18.2)

* No reactive cases for “other” gender; hence, gender-specific diagnostic yield is not determined in this scenario.

**Table 6:** HIV test results and diagnostic yield among participants, overall and by sex.

Sex	Test Result (n%)				Diagnostic Yield % (95% CI)
	Reactive	Non-Reactive	Invalid	Total	
Female	25	658	7	690	3.62 (2.46–5.2)
Male	22	664	8	694	3.17 (2.1–4.7)
* Other		3		3	-
Total	47	1325	15	1387	3.39 (2.5–4.4)

* No reactive cases for “other” gender; hence, gender-specific diagnostic yield is not determined in this scenario.

## Data Availability

The data provided in this manuscript is aggregate and the results of a descriptive analysis. Individual-level records are not available due to patient confidentiality. An aggregated dataset, or a limited dataset in which all cells have N > 5 can be made available upon reasonable request from the corresponding authors at cara.spence@usask.ca or stuart.skinner@usask.ca.

## Supplementary Materials

The following supporting information can be downloaded at: https://www.mdpi.com/article/10.3390/diagnostics16213445/s1. Figure S1: Mapping Study Sites for the Syphilis/HIV Early Rapid Response (SHIVER) Implementation Project in Saskatchewan, Canada.

## Abbreviations

The following abbreviations are used in this manuscript:

HIV	Human Immunodeficiency Virus
STIs	Sexually transmitted infections
POCT	Point-of-care testing
SHIVER	Syphilis/HIV Early Rapid Response
RE-AIM	Reach, Effectiveness, Adoption, Implementation, and Maintenance
AIDS	Acquired immunodeficiency syndrome
