# Supplementary material for: Syphilis/HIV Early Rapid Response (SHIVER): Community-Driven Point-of-Care Testing (POCT) Across Diverse Sites in Saskatchewan, Canada
Source: Diagnostics (Basel). 2026 Oct 22;16(21):3445. doi: 10.3390/diagnostics16213445 (PMC13762075; doi:10.3390/diagnostics16213445)
Supplement: Supplementary file 1 [file diagnostics-16-03445-s001.zip › diagnostics-4588965-supplementary.pdf]

Supplementary Materials:

Figure S1: Mapping Study Sites for the Syphilis/HIV Early Rapid Response (SHIVER) Implementation Project in Saskatchewan, Canada

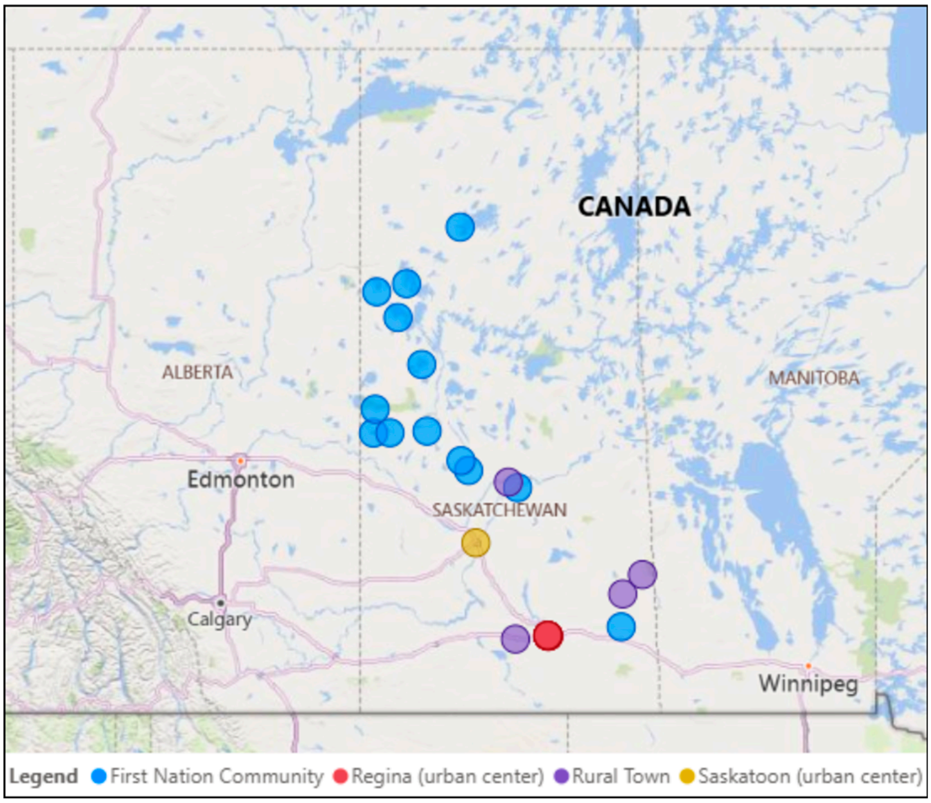

The study incorporated a diverse array of 28 sites across Saskatchewan, spanning urban centers, rural, and remote regions. Within rural and remote areas, the research engaged 14 First Nations communities and 4 rural towns. In the urban centers, the study involved one site in Saskatoon and nine sites in Regina.
